# Novel adjuvants from seaweed impede autophagy signaling in therapy-resistant residual pancreatic cancer

**DOI:** 10.1186/s12929-015-0132-4

**Published:** 2015-04-17

**Authors:** Sheeja Aravindan, Satish Kumar Ramraj, Somasundaram T Somasundaram, Natarajan Aravindan

**Affiliations:** Department of Marine Sciences, Center of Advanced Study in Marine Biology, Annamalai University, Parangipettai, TN India; Stephenson Cancer Center, Oklahoma City, OK USA; Department of Radiation Oncology, University of Oklahoma Health Sciences Center, Oklahoma City, OK USA

**Keywords:** Autophagy, Seaweed, Polyphenols, Pancreatic cancer, Marine brown algae, Pancreatic cancer relapse

## Abstract

**Background:**

Identifying the drug-deliverables that target autophagy is crucial to finding a cure for pancreatic cancer (PC), as activated autophagy is associated with poor patient outcomes. Our recent studies recognized the anti-PC potential of an antioxidant-rich collection of seaweed polyphenols and identified potential compounds for the treatment of PC. Accordingly, we investigated whether such compounds could regulate autophagy in therapy-resistant PC cells *in vitro* and in residual PC *in vivo*.

**Results:**

Human Panc-3.27 and MiaPaCa-2 cells were exposed to fractionated irradiation (FIR) with/without ethyl acetate (EA) polyphenol from *Spatoglossum asperum* (SA-EA), *Padina tetrastromatica* (PT-EA), or *Hormophysa triquerta* (HT-EA). The cells were subjected to QPCR to examine transcriptional alterations in the following autophagy functional regulators: *ATG3, ATG5, ATG7, ATG12, LC3A, LC3B, Beclin, Myd88, HMGB1, Rage*, and *TLRs 1-9*. Using a clinically relevant mouse model of residual PC, we use tissue microarray (TMA) and immunohistochemistry (IHC) procedures to investigate the potential of polyphenol(s) to target ATG3, ATG5, ATG12, LC3A, LC3B, BECN1, and SURIVIN after clinical radiotherapy. Radiation significantly increased the transcription of autophagy functional regulators in both cell lines. Seaweed polyphenols completely suppressed the transcription of all investigated autophagy regulators in both cell-lines. Gene silencing approach defined the role of LC3B in radiation-induced cell survival in this setting. TMA-IHC analysis revealed the complete regulation of ATG3, ATG5, ATG12, LC3A, LC3B, BECN1, and SURVIVIN in residual PC following SA-EA, PT-EA, and HT-EA treatment.

**Conclusions:**

These data demonstrate the autophagy blue print in therapy-resistant PC cells for the first time. Moreover, the data strongly suggest that the selected polyphenols could serve as effective adjuvants for current PC treatment modalities and may inhibit tumor relapse by comprehensively targeting therapy-orchestrated autophagy in residual cells.

## Background

Autophagy is the sequential process by which the cytoplasmic components are engulfed by phagophore, isolated in autophagosome, and broken down in the lysosome [1]. Autophagy, a well-regulated cell survival mechanism, plays a crucial role in the maintenance of normal human physiological processes, including cellular homeostasis, energetic balance, and development [2,3]. Conversely, autophagy is also implicated in the pathogenesis of many diseases, including cancer [4-8]. Autophagy, through its ability to shape inflammatory reactions [9], metabolic requirements [10], and oxidative stress [11], plays a multifaceted role in cancer [8,12-15], including increasing tumorigenesis and enhancing cancer development [16,17]. Compared with other epithelial cancers, pancreatic cancer (PC) generally exhibits high basal autophagy [18]. Activated autophagy is correlated with poor patient outcomes [19]. Primary therapeutic modalities for PC have been shown to galvanize autophagy [20-23]. Additional studies have recognized its cytoprotective role following chemo-radiotherapy [24,25]. Reactive oxygen species (ROS) toxicity, the mechanism upon which radiotherapy and some chemotherapy agents rely to eradicate tumor cells, has also been shown to regulate intracellular processes, including apoptosis and autophagy [26]. In this study, we investigated the autophagy-related molecular programming in human PC cells that survive first-line therapy.

The molecular orchestration of autophagy is complex and involves more than 30 autophagy-related genes (ATGs) [27,28]. Silencing these crucial autophagy drivers significantly inhibits cell growth and colony formation of PC cells [16]. Moreover, studies have shown that inhibition of autophagy augments the production of ROS, increased DNA damage, and regulates metabolism [16], suggesting the key role of autophagy in PC via the regulation of mitochondrial function and energy production. Identifying autophagy inhibitors that could be used in combination with chemotherapy, radiotherapy, and/or immunotherapy [29] may provide clinical benefits in the treatment of PC [18,16]. Recognizing a drug-deliverable that not only regulates basal elevated levels of autophagy, but also targets the therapy-associated onset, activation, and maintenance of autophagy would have momentous impact in the mitigation of PC relapse and metastasis, the major hurdles to curing PC. To our knowledge, no studies have attempted to examine potential of seaweeds or seaweed fractions (polyphenols) to inhibit autophagy in PC or other tumor models. This study investigates the autophagy-inhibiting potential of anti-oxidant rich seaweed polyphenol fractions.

The cells that survive first-line therapy exert immense adaptive resistance to the successive courses of therapy and further play crucial roles in the relapse, recurrence, and dissemination of disease to distant organs. As discussed above, the onset of autophagy-associated survival signaling has been demonstrated after primary therapeutic treatment, at least in PC. Identifying the adjuvants that mitigate the activation of autophagy-associated survival signaling in therapy-resistant cells could help us to cure PC. In the recent past, a number of epidemiological and experimental studies have attempted to identify the benefits of dietary habits, including the consumption of seaweeds, in the prevention of PC [30]. Seaweeds rich in polyphenols [31] have been shown to exert antitumor [32] potential, particularly in inhibiting cancer cell proliferation [33], tumor regression [34], and metastasis [35]. Recently, we have defined the anti-PC potential of polarity-based polyphenol extractions from five different seaweeds and further recognized the potential molecular targets in this setting [36]. Screening 20 polyphenol fractions in terms of their anti-oxidant capacity, and ability to inhibit cell viability, cell survival, DNA fragmentation, apoptosis, functional activation of NFκB, transcription/translation of key (Bcl2, PDGFA, AKT, kRAS, EGFR, VEGF, TERT, FGF) oncogenes and tumor progression molecules in genetically diverse PC cells identified three polyphenols (ethyl acetate fractions of *Hormophysa triquerta* HT, *Spatoglossum asperum* SA, *Padina tetrastromatica* PT) with superior anti-tumor activity. Accordingly, in this study, we used a clinically translatable residual PC model to investigate the clinical benefits of seaweed polyphenols in regulating the onset and maintenance of autophagy, particularly in the cells that survive first-line therapy. Achieving such benefits from the polarity gradient polyphenol fractions of seaweeds might identify potential adjuvant(s) that could be an effective modulator of autophagy in residual PC.

## Methods

### Cell culture

Genetically diverse human Panc-3.27 (ATCC-CRL2549) and MiaPaCa-2 (ATCC-1420) cells were obtained from Dr. Daniel J. Brackett (Department of surgery, University of Oklahoma Health Sciences Center, Oklahoma City, OK). Culture and maintenance of Panc-3.27 and MiaPaCa-2 cells were performed as described earlier [36,37]. For passage and for all experiments, the cells were detached using trypsin (0.25%)/EDTA (1%) resuspended in complete medium, counted electronically using a Countess automated cell counter (Carlsbad, CA, USA), and incubated in a 95% air/5% CO_2_ humidified incubator.

### Xenotransplantation mouse model

All experiments conformed to American Physiological Society standards for Animal Care and were carried out in accordance with the guidelines laid down by the National Research Council. Protocols were approved by our Institutional Animal Care and Use Committee before work began. Seven-week-old male athymic NCr-*nu/nu* nude mice (NCI, Frederick, MD) weighing 25-30 g were acclimatized for at least 3 days before the study. The mice were anesthetized using 0.2 ml of ketamine (10 mg/ml) and xylazine (1 mg/ml) in sterile phosphate buffered saline (PBS) via intraperitoneal injection, before the tumor xenograft and irradiation procedures. We subcutaneously administered 5×10^6^ human Panc-3.27 or MiaPaCa-2cells suspended in 30% Matrigel (BD Biosciences) into the animals’ right flanks. Tumor growth was periodically monitored. Tumors were allowed to grow to a volume of up to 100 mm^3^. Six animals were used per treatment group, and animals were randomly allocated to each group [38-42]. Animals were monitored and checked by veterinary staff daily, and weighed twice a week. All efforts were made to minimize and alleviate animal discomfort. An animal was considered to be in distress if it experienced any of the following: loss of 25% or more body weight, unthriftiness (inability to walk, run, eat or drink properly) due to injury or potential tumor load, became moribund and lethargic, or developed visible and/or necrotic tumors. Mice that displayed bleeding, wounds, any other symptoms that affected posture, or appeared to be uncomfortable were sacrificed early. At the end of each experiment, animals were euthanized by CO_2_ asphyxiation. Xenografts were harvested and subjected to downstream endpoint analysis.

### *In vitro* and *In vivo* irradiation procedures

In the radiation experiments, Panc-3.27 or MiaPaCa-2 cells were either mock-irradiated or exposed to FIR (2 Gray (Gy)/day for 5 days) using a Gamma Cell 40 Exactor (Nordion International Inc., Ontario, Canada) at a dose rate of 0.81 Gy/min. *In vivo* PC xenografts established from corresponding PC cells were selectively exposed to clinically relevant FIR (2 Gy/day for 5 days/week for a total of 3 weeks) to a total dose of 30 Gy. A specially designed cerrobend shield was used to encase the body of the mice, exposing only the flank tumors, as described earlier [43-45]. Mock-irradiated animals were treated identically, except that they were not subjected to radiation. For all *in vivo* experiments we used a tumor (xenograft) focused clinically relevant fractionated radiation dose regimen (2Gy/Day for 5 days/week for three weeks with a total dose of 30Gy). In each case, FIR alone group or with the seaweed polyphenol treatments, we started radiotherapy as early as the tumor reaches 100 mm^3^. This approach particularly allowed us to establish a clinically relevant residual tumor model, avoiding any bulk tumor with the possibility of tumor mass/cells that spared out of radio-therapeutic field. At the end of complete radiation dose regimen with or without seaweed polyphenol(s) treatment, the tumor xenografts were maximally reduced, residual and beyond any unbiased comparable measure between groups. Hence, tumor growth measurements in response to radiotherapy as well as with the seaweed polyphenols are not included in this study.

### Polarity gradient polyphenol(s) extraction and cell treatments

Cells were plated in 100 mm tissue culture plates containing 6 ml of complete growth medium and were allowed to grow up to 80% confluence. Polarity-based extractions of seaweed polyphenol fractions were performed as described earlier [36]. We selectively examined ethyl acetate fractions of *Hormophysa triquerta* (HT), *Spatoglossum asperum* (SA), and *Padina tetrastromatica* (PT) those demonstrated superior anti-PC activity measured in terms of their ability to inhibit cell viability, cell survival, DNA fragmentation, apoptosis, functional activation of NFκB, transcription/translation of key (Bcl2, PDGFA, AKT, kRAS, EGFR, VEGF, TERT, FGF) oncogenes and tumor progression markers [36]. For all *in vitro* investigations, cells were treated with 100 μg/ml of each polyphenol fraction, while corresponding 10 mg/Kg concentration was used for *in vivo* studies.

### Quantitative Real-time PCR

Total RNA extraction and individual gene transcriptional levels were investigated as described earlier [46,47]. Transcriptional alterations of *ATG3, ATG5, ATG7, ATG12, TLR-1, TLR-2, TLR-3, TLR-4, TLR-5, TLR-6, TLR-7, TLR-8, TLR-9, HMGB-1, RAGE, LC3A, LC3B, Beclin*, and *MYD88* were investigated. For this, we adopted two approaches: (i) PC cells pre-treated with select seaweed polyphenol were then exposed to fractionated irradiation and, (ii) cells exposed to fractionated irradiation were treated with seaweed polyphenol. We used β-actin as a positive control. A negative control without template RNA was also included. Each experiment was carried out in triplicate. The ΔΔ^Ct^ values were calculated by normalizing the gene expression levels to β-actin. The relative expression level was expressed as fold change. Group-wise comparisons were made using ANOVA with Tukey’s post-hoc correction. A *P* value of <0.05 was considered statistically significant.

### Bromodeoxyuridine (BrdU)-incorporation cell proliferation assay

Serum starved MiaPaCa-2 or Panc-3.27 cells that were either mock-irradiated, exposed to fractionated irradiation (2Gyx5), treated with SA-EA, PT-EA or SA-EA, treated with SA-EA, PT-EA or SA-EA and exposed to FIR, exposed to FIR and treated with SA-EA, PT-EA or SA-EA or LC3B silenced and exposed to mock-IR or FIR were transferred to 96-well plates (2x10^3^/well). Twenty-four hours after stimulation with FBS, cells were treated with BrdU (1 μM) for 2 h, washed and fixed in sucrose supplemented 3% paraformaldehyde in PBS. Fixed cells were serially treated with 0.1% triton buffer and 2 M HCl with excessive intermittent PBS washing. The cells were then blocked (0.1% BSA in PBS), tagged with anti-BrdU mouse antibody (for 1 h at 37°C), washed and second labelled with goat-anti mouse Alex Flour-488. Unlabeled nucleus were then stained with DAPI and analyzed in Operetta high content confocal imaging. BrdU incorporation was captured for 63 different fields/well with three different Z planes. Quantification of the total and BrdU incorporated nuclei are counted using Operetta integrated automated Columbus image analysis software. Group-wise comparisons were computed using GraphPad PRISM and analyzed using Two-way ANOVA with Tukey’s post-hoc correction.

### Plasmid preparation and DNA transfection

To define the role of autophagy signaling in the proliferation of PC cells that survive radiotherapy, we inhibited LC3B, the well documented autophagic marker, the amount of which correlates with the amount of autophagic membranes and examined for the alterations in cell proliferation. Transient transfection of LC3B shRNA (MISSION® shRNA, Sigma-Aldrich) in human MiaPaCa-2 and Panc-3.27 cells were carried out by using either TurboFectin 8.0 reagent (Origene) or Neon electroporation transfection system (Life Technologies).

### TMA construction and immunohistochemistry/immunofluorescence

All TMA construction procedures and automated IHC were performed in the Charles Stephenson Cancer Center Cancer Tissue Pathology Core. Briefly, harvested PC xenografts were fixed in 10% buffered formaldehyde, embedded in paraffin, and used to extract individual tissue cores. All H&E stained slides were reviewed for pathology. We selected the most representative areas of tumor cells for tumor sampling. We examined TMA constructed from residual PC xenografts after clinical doses of fractionated radiotherapy for cellular localization and expression of survivin (Santa Cruz), LC3A, LC3B, ATG3, ATG5, ATG12, and Beclin1 (Cell Signaling Technology Inc., Danvers, MA, USA). Appropriate tissue morphologic/pathologic (Hematoxylin and Eosin, H&E) controls, positive expression controls, and negative (no primary antibody (Ab) controls were examined in parallel. The slides were micro-digitally scanned using a Scanscope (Aperio) slide scanner and analyzed using integrated Spectrum software. For Beclin1 (Santa Cruz), the primary protein was tagged with Alexa Fluor 488 (Abcam), while the cell membrane was marked with wheat germ agglutinin (WGA) Alexa Fluor 594 (Invitrogen) and nuclear counterstained with DAPI. Immunofluorescence was measured with the high-throughput optical imager Operetta (Perkin Elmer) and analyzed with the integrated Columbus image data storage and analysis system (Perkin Elmer).

## Results

### Activated autophagy machinery in resistant PC cells after first-line Radiotherapy

**Figure 1:**
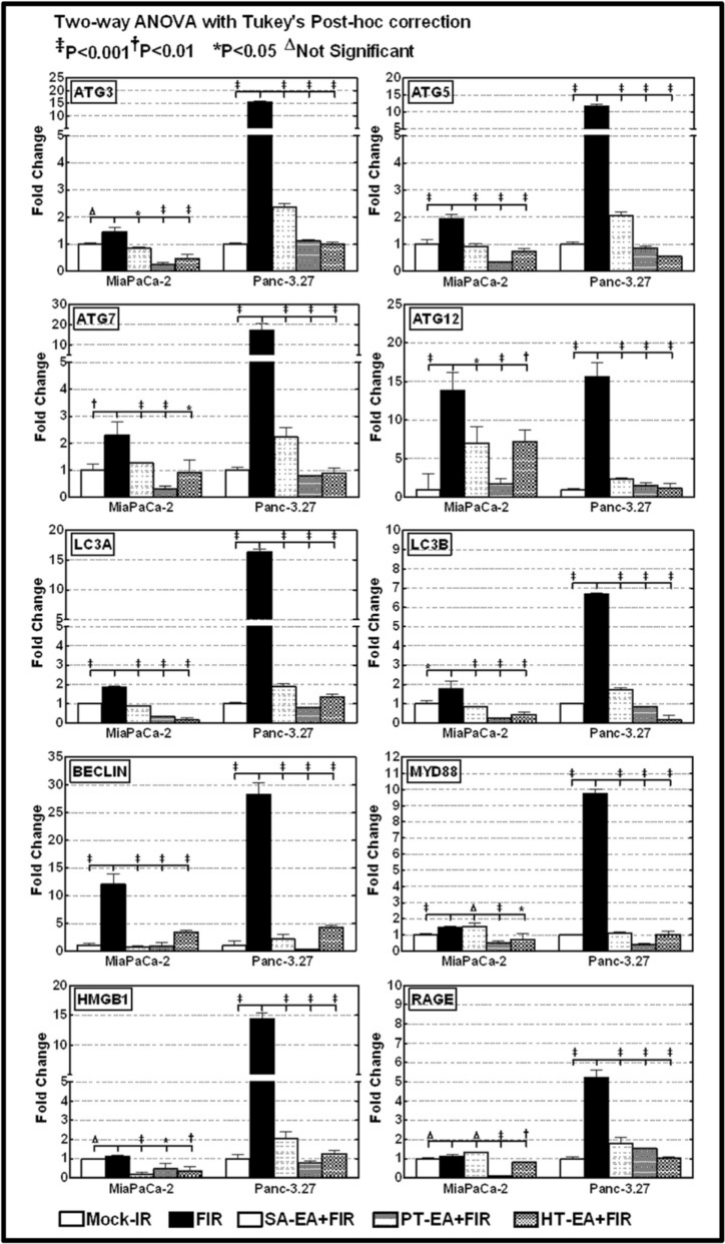
Individual gene QPCR analysis showing transcriptional modulations of *ATG3, ATG5, ATG7, ATG12, LC3A, LC3B, Belcin1, MyD88, HMGB1*, and *RAGE* in Panc-3.27 and MiaPaCa-2 cells, either mock-irradiated, exposed to FIR (2Gy x 5d) or *pretreated with 100* μ*g/ml of SA-EA, PT-EA, or HT-EA seaweed polyphenol fractions and exposed to FIR.* Pretreating the cells with seaweed polyphenols significantly attenuated radiotherapy-induced *ATG3, ATG5, ATG7, ATG12, LC3A, LC3B, Belcin1, MyD88, HMGB1*, and *RAGE* transcription in Panc-3.27 and MiaPaCa-2 cells.

**Figure 2:**
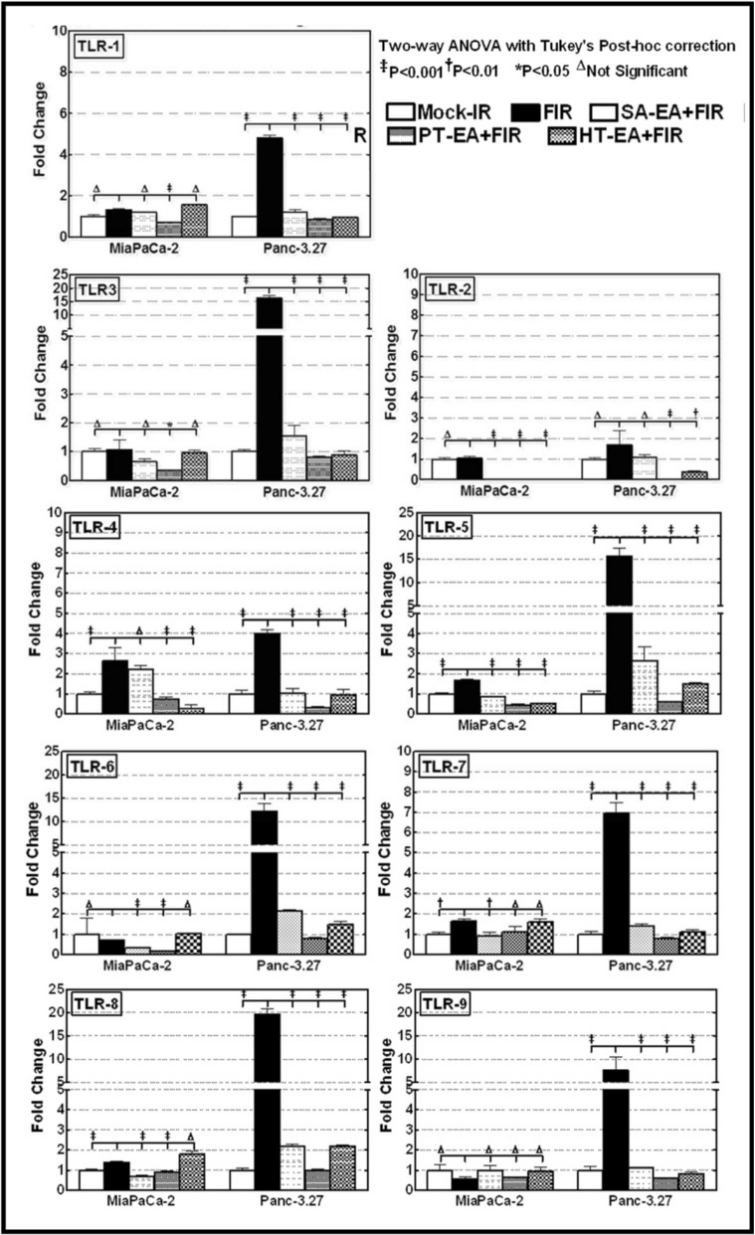
Individual gene QPCR analysis showing transcriptional modulations of TLRs (TLR 1-9) in Panc-3.27 and MiaPaCa-2 cells, either mock-irradiated, exposed to FIR (2Gy x 5d) or pretreated with 100 μg/ml of SA-EA, PT-EA, or HT-EA seaweed polyphenol fractions and exposed to FIR. Pretreating the cells with seaweed polyphenols significantly attenuated radiotherapy-induced TLRs 1-9 in transcription in Panc-3.27 and MiaPaCa-2 cells.

To better characterize the autophagy phenotype of the PC cells that survive first-line therapy, we examined the transcriptional modulation of autophagy functional regulators *ATG3, ATG5, ATG7, ATG12, TLR-1, TLR-2, TLR-3, TLR-4, TLR-5, TLR-6, TLR-7, TLR-8, TLR-9, HMGB-1, RAGE, LC3A, LC3B, Beclin*, and *MYD88*, in genetically diverse human Panc-3.27 and MiaPaCa-2 cells exposed to clinically relevant fractionated radiation (2 Gy/Day for 5 days). Real-time QPCR revealed a significant (*P* < 0.001) induction of *ATG3, ATG5, ATG7, ATG12, HMGB-1, RAGE, LC3A, LC3B, Beclin, MYD88* (Figure 1), *TLR-1, TLR-2, TLR-3, TLR-4, TLR-5, TLR-6, TLR-7, TLR-8,* and *TLR-9* (Figure 2) in both cell lines after FIR. We observed a robust activation of these molecules in Panc-3.27 cells compared with MiaPaCa-2 cells. Although the activated transcription was evident in MiaPaCa-2 cells, the activation of *ATG3, HMGB1, RAGE, TLR-1, TLR-2, TLR-3, TLR-6* and *TLR-9* were marginal after FIR. Therapy-associated activation of these molecules, except *TLR-2*, was profound (>10 fold) in Panc-3.27 cells. Activation of these functional regulators of autophagy and its signal transduction flow through demonstrates the intrinsic influence of the autophagy-associated survival response in therapy-resistant PC cells.

### Seaweed polyphenols target therapy-orchestrated autophagy transcriptional machinery in surviving PC cells

To determine the potential of seaweed polyphenols in targeting radiotherapy-induced regulation of autophagy in surviving PC cells, we examined the transcriptional modulation of *ATG3, ATG5, ATG7, ATG12, TLR-1, TLR-2, TLR-3, TLR-4, TLR-5, TLR-6, TLR-7, TLR-8, TLR-9, HMGB-1, RAGE, LC3A, LC3B, Beclin*, and *MYD88* in Panc-3.27 and MiaPaCa-2 cells. Cells were pretreated with SA-EA, PT-EA, and HT-EA fractions and exposed to radiotherapy (2 Gy x 5 days). Therapy-associated activation of *ATG3* was significantly inhibited in both cells lines in the presence of SA-EA, PT-EA, and HT-EA fractions (Figure 1). All three fractions consistently induced *cell-line independent* inhibition of FIR-induced *ATG5* transcription in PC cells (Figure 1). PT-EA prompted maximum inhibitory efficiency of *ATG5* in MiaPaCa-2 cells, while HT-EA showed more efficiency in Panc-3.27 cells. Moreover, we observed significant inhibition of therapy-associated *ATG7* transactivation in both cell lines with SA-EA, PT-EA, and HT-EA treatment (Figure 1). Further, QPCR analysis recognized the definite inhibition of radiotherapy-induced *ATG12* transactivation in PC cells in the presence of seaweed polyphenols (Figure 1), with maximum efficiency of PT-EA in MiaPaCa-2 and HT-EA in Panc-3.27 cells. Autophagic flux markers *LC3A* and *LC3B* were significantly activated in response to therapy, and were completely suppressed in PC cells treated with SA-EA, PT-EA, or HT-EA fractions (Figure 1). Though we observed marginal variations in *LC3A* and *LC3B* inhibition between the polyphenol fractions, all three polyphenols inflicted maximal (*P* < 0.001) transcriptional repression of these two genes. These high polarity-based extractions of seaweeds also demonstrated a *cell-line independent* inhibition of autophagy signaling genes, including *HMGB1, RAGE* and *Myd88*, in radio-resistant PC cells (Figure 1). Similarly, all three fractions imposed significant *cell-line independent* inhibition of FIR-induced *Beclin (ATG6)* transcription in PC cells (*P* < 0.001, Figure 1). SA-EA induced maximum inhibitory efficiency in MiaPaCa-2 cells, while PT-EA showed more efficiency in Panc-3.27 cells.

Since Toll-like receptors (TLRs) are intrinsically involved in micro-autophagy and have been shown to be activated in response to therapy in many tumor models, we investigated the potential of seaweed polyphenols in the regulation of therapy-orchestrated TLR signaling in macroautophagy. QPCR analysis revealed a marked decrease in the radiotherapy-induced *TLRs-1, -2, -3,-4, -5, -6, -7, -8*, and *-9* in MiaPaCa-2 and Panc-3.27 cells (Figure 2). All fractions exhibited similar inhibitory profiles with marginal variations. Though there was no notable increase in *TLR-2* after therapy, the fractions defined the inhibition of constitutive TLR2 in this setting. Taken together, the results recognized the regulation potential of SA-EA, PT-EA, and HT-EA fractions in the autophagy machinery that drives the survival of PC cells after therapy.

**Figure 3:**
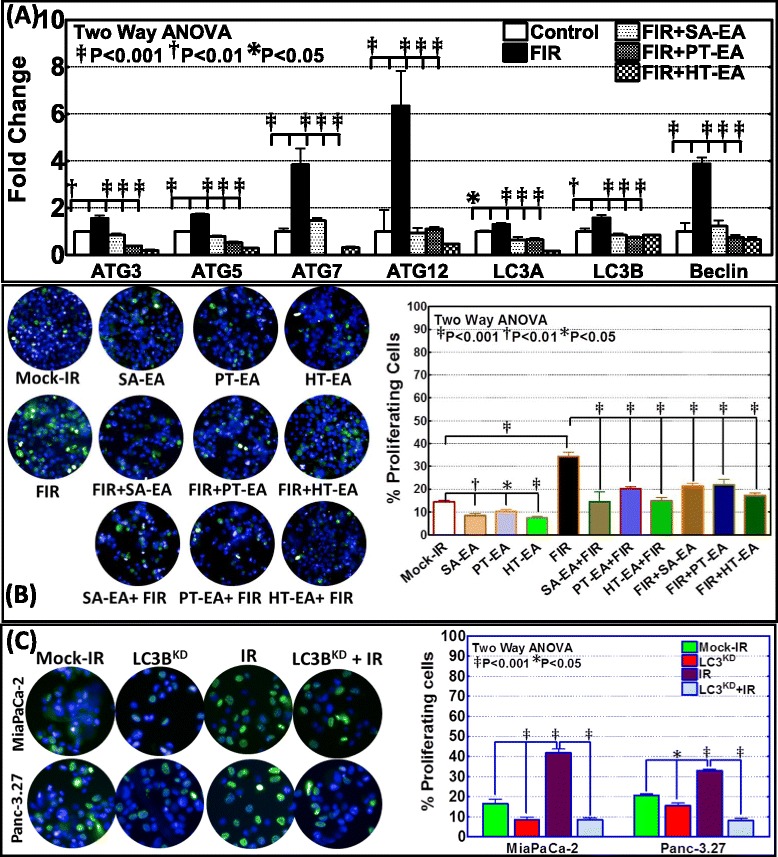
Seaweed polyphenols exerts autophagy dependent anti-tumor activity and promotes radio-sensitization/radio-protection in PC cells. **(A)** Individual gene QPCR analysis showing transcriptional modulations of *ATG3, ATG5, ATG7, ATG12, LC3A, LC3B* and *Belcin1* in MiaPaCa-2 cells, either mock-irradiated, exposed to FIR (2Gy x 5d) *or exposed to FIR and treated with 100* μ*g/ml of SA-EA, PT-EA, or HT-EA seaweed polyphenol fractions.* Treating the radio-resistant cells with seaweed polyphenols significantly inhibited *ATG3, ATG5, ATG7, ATG12, LC3A, LC3B* and *Belcin1* transcription in radio-resistant Panc-3.27 and MiaPaCa-2 cells. **(B)** Representative images of Operetta high-throughput confocal imaging showing BrdU incorporation levels in MiaPaCa-2 cells either mock irradiated, exposed to fractionated irradiation (FIR, 2Gy x 5d), exposed to seaweed polyphenols (SA-EA, PT-EA or HT-EA - 100 g/ml)**,** exposed to each seaweed polyphenols followed by FIR or exposed to FIR followed by seaweed polyphenol treatment. Histograms obtained from Operetta integrated automated Columbus image analysis software computed BrdU incorporation for 63 different fields/well showing significant reduction in radiotherapy-induced proliferating cells with seaweed polyphenol treatment. **(C)** Representative images of Operetta high-throughput confocal imaging showing BrdU incorporation levels in MiaPaCa-2 and Panc-3.27 cells either mock irradiated, exposed to radiation (2Gy), LC3B silenced or LC3B silenced and exposed to IR. Histograms obtained from automated Columbus image analysis computed BrdU incorporation (for 63 different fields/well) showing significant reduction in radiotherapy-activated cell proliferation with LC3B silencing.

Further, to determine the post-radiation (radio-protection) benefit of seaweed polyphenol in the regulation of autophagy transcriptional machinery, if any, we examined the transcriptional modulation of *ATG3, ATG5, ATG7, ATG12, LC3A, LC3B* and *Beclin* in MiaPaCa-2 cells. Cells exposed to FIR were then treated with SA-EA, PT-EA, and HT-EA fractions. Radiation-induced activation of *ATG3* was significantly inhibited in the presence of SA-EA, PT-EA, and HT-EA fractions (Figure 3A). All three fractions consistently inflicted inhibition of FIR-induced *ATG5* transcription (Figure 3A). Moreover, we observed significant inhibition of radiation-associated *ATG7* transactivation with SA-EA, PT-EA, and HT-EA treatment (Figure 3A). Further, QPCR analysis recognized the definite inhibition of radiotherapy-induced *ATG12* transactivation in PC cells in the presence of seaweed polyphenols. Autophagic flux markers *LC3A* and *LC3B* that were significantly activated in response to radiotherapy were completely suppressed in MiaPaCa-2 cells treated with SA-EA, PT-EA, or HT-EA fractions (Figure 3A). Similarly, all three fractions imposed significant inhibition of FIR-induced *Beclin (ATG6)* transcription (Figure 3A). Together, these date demonstrate that treatment of radio-resistant PC cells with seaweed polyphenols exhibit significant inhibition of autophagy transcriptional machinery implying the possible benefit of these fractions in autophagy dependent radioprotection of surviving PC cells.

### Seaweed polyphenols exercise anti-tumor activity and promotes radio-sensitization/radio-protection in PC cells

Number of studies has recognized the activation of proliferation/clonal expansion capabilities in tumor cells that survive after fractionated radiation [48,49]. Further, we demonstrated the activation NFκB-dependent survival signaling after fractionated irradiation in multifarious tumor models [50,51] including pancreatic cancer [37]. To determine the benefit of the seaweed polyphenols in PC cell radio-sensitization and/or radio-protection, we adopted three approaches. First, MiaPaCa-2 cells treated with SA-EA, PT-EA or HT-EA as stand-alone compounds were examined for their anti-tumor activity. Compared to the mock-irradiated controls, BrdU incorporation assay revealed a significant inhibition of PC cell proliferation with SA-EA (P < 0.01), PT-EA (P < 0.05) and HT-EA (P < 0.001) treatment (Figure 3B). Next, MiaPaCa-2 cell pre-treated with seaweed polyphenols were exposed to FIR and examined for their radio-sensitization potential. Parallel FIR exposed cells were used as controls. Compared to the mock-irradiated cells, we observed a significant activation of cell proliferation in cells that survive FIR. Conversely, cells pretreated with SA-EA, PT-EA or HT-EA and exposed to FIR revealed a complete (P < 0.001) suppression of FIR-associated increase in cell proliferation (Figure 3B) demonstrating the radio-sensitizing potential of all three seaweed polyphenols investigated. Finally, MiaPaCa-2 cell exposed to fractionated irradiation were then treated with SA-EA, PT-EA or HT-EA and examined for their radio-protection potential. Interestingly, treating the radio-resistant cells with seaweed polyphenols significantly (P < 0.001) reduced the cell-proliferation capacity implying their radio-protecting benefit in this setting (Figure 3B). Together, these data corroborates well with the autophagy transcriptional machinery alterations data and identify the novel radio-sensitizing as well as radio-protecting potential of seaweed polyphenols.

### Autophagy mediates cell proliferation in radio-resistant cells

Further to define the role of autophagy on activated cell proliferation in PC cells that survive radiotherapy, LC3B silenced MiaPaCa-2 and Panc-3.27 cells were exposed to mock-IR or 2Gy radiation and analyzed for alterations in cell proliferation. Compared to the mock-irradiated controls, BrdU incorporation assay demonstrated a significant decrease in cell proliferation in LC3B muted MiaPaCa-2 (P < 0.001) and Panc-3.27 (P < 0.05) cells (Figure 3C). Cells that survive the radiation exposure exhibited robust (P < 0.001) proliferation rate. More importantly, the increased proliferation in cells that survive radiation was significantly attenuated in both MiaPaCa-2 and Panc-3.27 cells when autophagy marker, LC3B was silenced (Figure 3C). Together, these results demonstrate that onset of autophagy mediates activated cell proliferation in therapy resistant PC cells.

### SA-EA, PT-EA, and HT-EA fractions regulate ATG3, ATG5, ATG12, survivin, LC3A, LC3B, and beclin expression and localization in residual tumors *in vivo*

**Figure 4:**
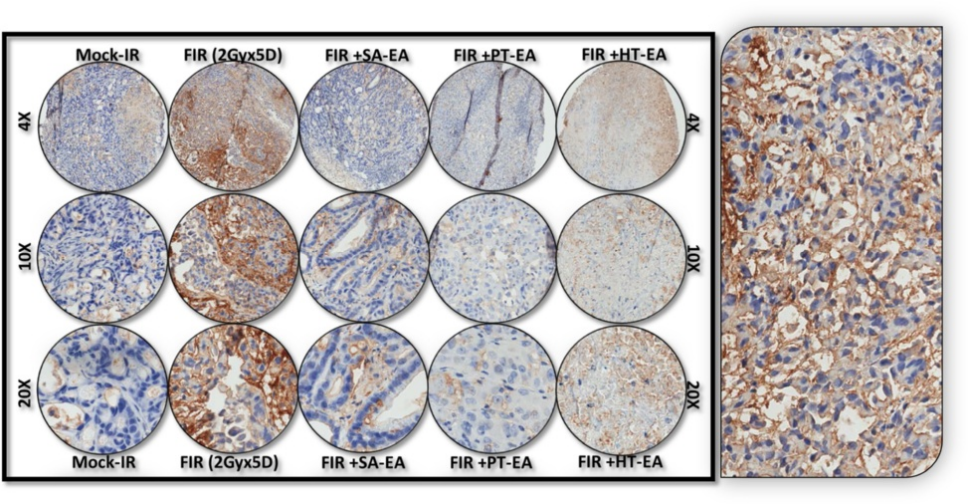
Representative microphotographs from *ATG3-*stained PC tissue micro array (TMA) constructed with xenografts (established from *MiaPaCa-2*) exposed to mock-irradiation or fractionated irradiation with or without SA-EA, PT-EA, and HT-EA fractions. *Pullout* shows the staining pattern (20x magnification).

**Figure 5:**
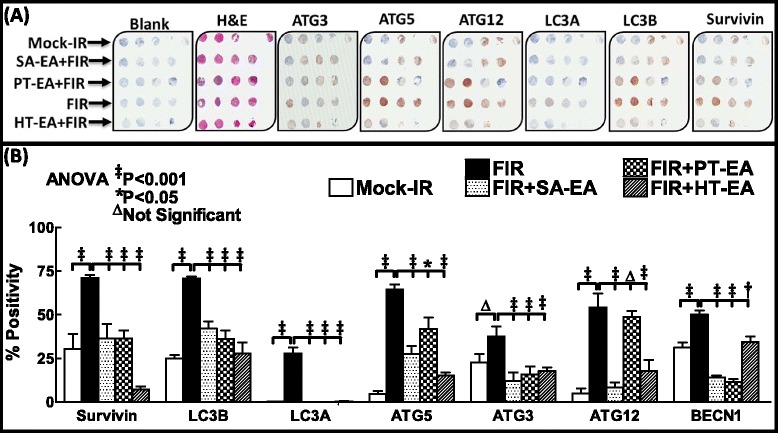
Seaweed polyphenol treatments regulate autophagy related (ATG3, ATG5, ATG12, LC3A, LC3B, Survivin ) protein expression in radiotherapy resistant residual PC tumors. **(A)** Images of TMAs labelled (automated IHC) for Blank (no primary antibody), hematoxylin-eosin, ATG3, ATG5, ATG12, LC3A, LC3B, and survivin. **(B)** Aperio TMA-quantitation analysis showing protein specific positivity magnitudes in mock-irradiated PC xenografts and residual PC tissues after clinical radiotherapy with or without SA-EA, PT-EA, or HT-EA treatment. Group-wise comparisons were made using Two-way ANOVA with Bonferroni’s post-hoc correction.

To further substantiate that ethyl acetate fraction of SA, PT, and HT polyphenols regulate crucial and defined autophagic players that functionally orchestrate PC survival after first-line therapy, we examined the cellular expression levels of ATG3, ATG5, ATG12, survivin, LC3A, LC3B, and beclin in residual human PC tumors. We selectively exposed human PC xenografts established with MiaPaCa-2 cells in athymic nude mice to FIR (2 Gy/day for 5 days/week for three weeks) to a total dose of 30 Gy, with or without a daily dose of SA-EA, PT-EA, or HT-EA. To avoid IHC staining inconsistencies across samples, TMA was constructed with the xenografts and the protein localizations were examined with automated IHC coupled with micro digital scanning (Aperio) and spectrum analysis. TMA cores were carefully constructed utilizing histopathological evaluations of individual H&E stained tumor tissues and further confirmed with TMA-H&E staining. IHC staining analysis of autophagocytosis-associated protein ATG3 revealed basal levels of positivity in mock-irradiated xenografts. ATG3 positive staining appeared in brown, and was predominantly localized in the cytoplasm (Figure 4 pullout). Compared with mock-irradiated controls, ATG3 immunoreactivity was intense and present in 37.511 ± 5.69% of the PC cells in residual tumors after clinical doses of FIR (Figure 5). Conversely, we observed a complete loss of ATG3 immunoreactivity in tumors from mice that received SA-EA and were exposed to FIR. ATG3 immunoreactivity was completely reduced to basal (mock-IR) or lower levels in all residual tumors from animals treated with PT-EA in conjunction with radiotherapy (Figure 5B). Likewise, HT-EA treatment in combination with radiotherapy produced a complete inhibition of ATG3 cellular localization in residual PC tumors. SA-EA exhibited profound ATG3 inhibition in residual PC tumors after first-line radiotherapy.

**Figure 6:**
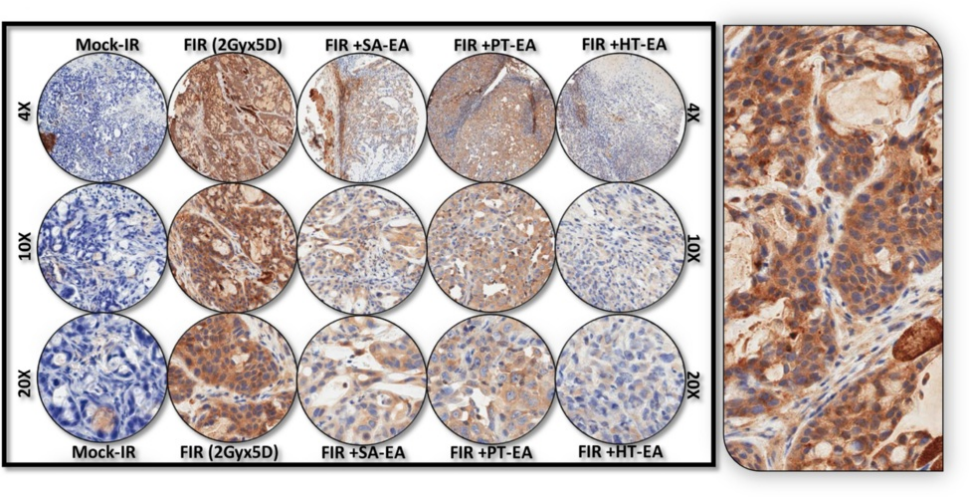
Representative microphotographs from *ATG5-*stained PC TMA constructed with xenografts (established from *MiaPaCa-2*) exposed to mock-irradiation or fractionated irradiation, with or without SA-EA, PT-EA, and HT-EA fractions. *Pullout* shows the staining pattern (20x magnification).

**Figure 7:**
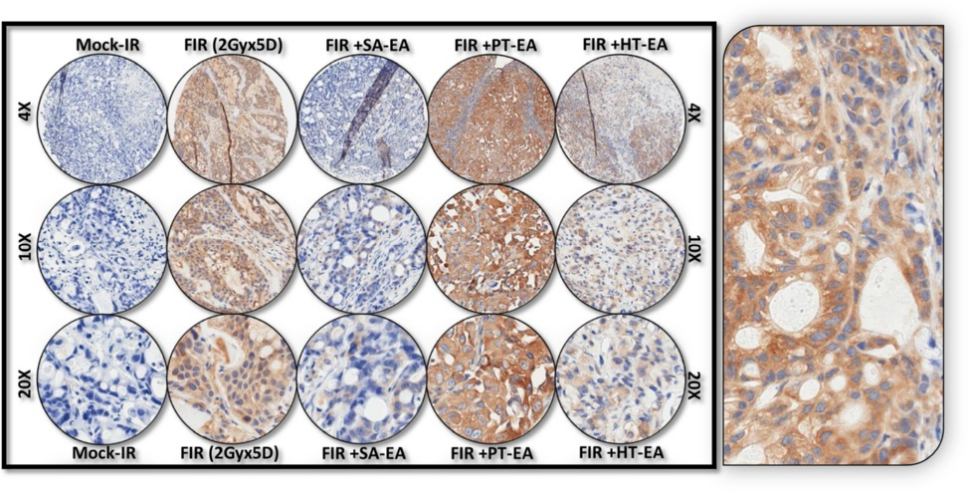
Representative microphotographs from *ATG12-*stained PC TMA constructed with xenografts (established from *MiaPaCa-2*) exposed to mock-irradiation or fractionated irradiation, with or without SA-EA, PT-EA, and HT-EA fractions. *Pullout* shows the staining pattern (20x magnification).

Figure 6 shows the representative results of IHC for autophagocytosis-associated protein ATG5 in PC xenografts that were either mock-irradiated or exposed to clinical FIR, with or without SA-EA, PT-EA, or HT-EA treatment. There was no detectable immunoreactivity with the negative control (data not shown). ATG5 expression was stronger in tumor cells than in stromal cells (Figure 6 pullout). Though ATG5 was detectable in all analyzed xenografts, radiotherapy-treated xenografts showed strong ATG5 expression. There was a significant difference of ATG5 expression between FIR-only and FIR with seaweed polyphenol(s) groups (Figure 5B). SA-EA and PT-EA treatments showed low levels of ATG5, compared with the FIR group. ATG5 expression was barely detectable with HT-EA treatment. All selected specimens of PC xenograft tissues contained ATG12–positive cells. Stained cytoplasm appeared dark brown in color and was mainly present in epithelial cells (Figure 7 pullout). ATG12 positivity ranged from 4.84 ± 2.65% of mock-irradiated xenografts to 54.098 ± 8.08% of irradiated residual tumors (Figure 5B). The range for ATG12–positive epithelial cells was <10% in animals that received SA-EA in conjunction with radiotherapy. However, the mean fraction of ATG12–positive nuclei was 48.668 ± 3.23% in xenografts of animals that received radiotherapy and PT-EA. HT-EA treatment yielded about 17.718 ± 6.09% ATG12–positive epithelial cells (Figure 5B).

**Figure 8:**
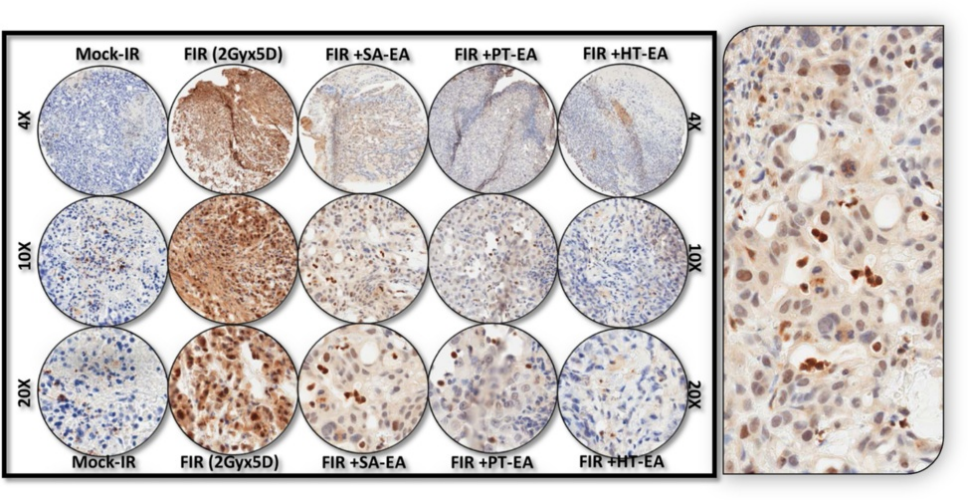
Representative microphotographs from *survivin-*stained PC TMA constructed with xenografts (established from *MiaPaCa-2*) exposed to mock-irradiation or fractionated irradiation, with or without SA-EA, PT-EA, and HT-EA fractions. *Pullout* shows the staining pattern (20x magnification).

**Figure 9:**
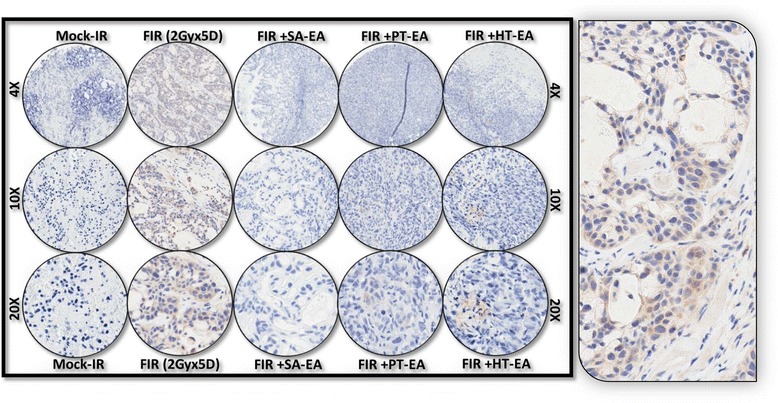
Representative microphotographs from *LC3A-*stained PC TMA constructed with xenografts (established from *MiaPaCa-2*) exposed to mock-irradiation or fractionated irradiation, with or without SA-EA, PT-EA, and HT-EA fractions. *Pullout* shows the staining pattern (20x magnification).

IHC staining of survivin revealed basal levels of positivity in mock-irradiated xenografts. The positive staining of survivin appeared in dark brown, and was localized in both the cytoplasm and the nucleus (Figure 8 pullout). There was no staining observed with the negative control for survivin (data not shown). Compared with the mock-irradiated controls, survivin immunoreactivity was intense and present in 70.998 + 1.621% of the PC cells in residual tumors after clinical doses of FIR (Figure 5). However, we observed a significant (*P* < 0.001) reduction in survivin immunoreactivity in tumors of mice that received SA-EA or HT-EA and were exposed to FIR. Figure 9 shows representative results of IHC for microtubule-associated protein 1 light chain 3 (LC3A) in PC xenografts that were either mock-irradiated or exposed to clinical FIR, with or without SA-EA, PT-EA, or HT-EA. We observed no detectable immunoreactivity of LC3A in the mock-irradiated group. In xenografts exposed to clinical radiotherapy, we observed a strong detectable LC3A immunoreactivity that was predominantly localized in the cytoplasm (Figure 9 pullout). PC xenografts from animals that received radiotherapy in conjunction with SA-EA showed little to no detectable cytoplasmic LC3A. Similarly, we did not see any detectable LC3A immunoreactivity in tumors of animals that received radiotherapy in combination with PT-EA or HT-EA (Figure 5B).

**Figure 10:**
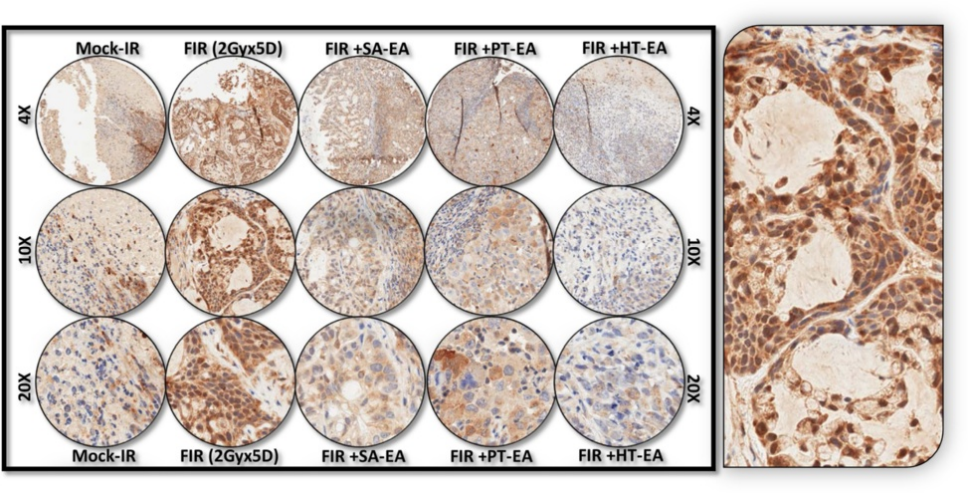
Representative microphotographs from *LC3B-*stained PC TMA constructed with xenografts (established from *MiaPaCa-2*) exposed to mock-irradiation or fractionated irradiation, with or without SA-EA, PT-EA, and HT-EA fractions. *Pullout* shows the staining pattern (20x magnification).

**Figure 11:**
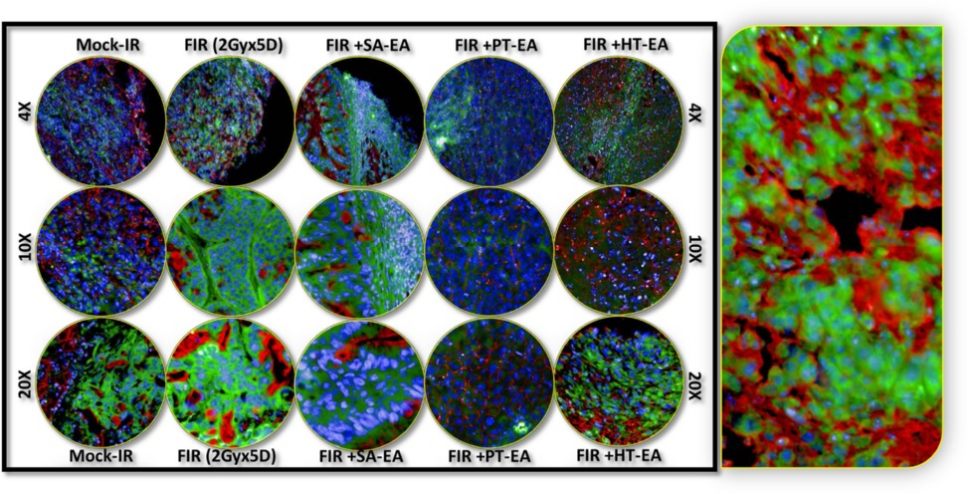
Representative microphotographs from *Beclin1-*stained PC TMA constructed with xenografts (established from *MiaPaCa-2*) exposed to mock-irradiation or fractionated irradiation, with or without SA-EA, PT-EA, and HT-EA fractions. *Pullout* shows the staining pattern (20x magnification).

We examined cellular localization levels of microtubule-associated protein 1 light chain 3B (LC3B) in human PC xenografts after radiotherapy in conjunction with SA-EA, PT-EA, or HT-EA treatment (Figure 10). LC3B immunoreactivity was detectable in mock-irradiated human PC xenografts, consistent with the comparable levels reported in human PC (www.proteinatlas.org). However, the tumors surviving after clinical radiotherapy exhibited high immunoreactivity for LC3B localization (70.663 ± 0.998%). LC3B positive staining appeared in brown, and was predominantly localized in the cytoplasm in a punctuated pattern (Figure 10 pullout). Regulation of the radiotherapy-induced localization of LC3B was evident in PC tissues from animals that received SA-EA, PT-EA, and HT-EA treatment (Figure 5). HT-EA treatment showed the high regulatory potential of radiotherapy-associated LC3B localization. We also examined beclin-1, a mammalian ortholog of the yeast autophagy-related gene 6 (BECN1) protein that has been shown to have relatively high levels of localization in PC tissues (www.proteinatlas.org), in human PC xenografts after radiotherapy, with or without treatment with seaweed polyphenol fractions (Figure 11). Mock-irradiated PC tumors showed detectable BECN1 immunoreactivity. Immunofluorescence was highly intense and present in 49.982 ± 3.284% of the PC cells in residual tumors after clinical FIR (Figure 5B). BECN1 positive staining appeared in green (Alexa Fluor 488), and was predominantly localized in cytoplasm (Figure 11 pullout). Interestingly, radiotherapy combined with SA-EA, PT-EA, or HT-EA completely regulated BECN1 localization in residual PC tumors, compared with those tumors receiving radiotherapy alone. HT-EA treatment in conjunction with radiotherapy significantly attenuated the FIR-induced cellular localization of BECN1 in this setting. Together, these data clearly portray the clinical benefit of SA-EA, PT-EA, and HT-EA fractions in the regulation of radiotherapy-orchestrated cellular localization of PC progression, driving survivin, ATG3, ATG5, ATG12, LC3A, LC3B, and beclin in residual PC tumors.

## Discussion

The role of the autophagic process in antitumor therapy has not been clearly established [52,53]. After treatment with antitumor drugs and/or radiation, some cancer cells undergo autophagy as a temporary survival mechanism. The suppression of autophagy leads to apoptosis, thus enhancing antitumor effects. There are three primary treatments for patients with exocrine PC: surgery, radiation therapy, and chemotherapy. Autophagy can be activated by chemotherapy and ionizing radiation in the treatment of PC cells [20-23]. The cytoprotective role of autophagy following chemotherapy and radiation therapy has been confirmed by many investigators [25]. In the present study using PC cells, we found that seaweed polyphenols targeted autophagy that was activated in response to first-line radiotherapy. Gemcitabine, as first-line therapy for patients with advanced PC, triggers autophagy in PC cells. Suppression of autophagy has been shown to promote apoptotic cell death in response to gemcitabine in PC [25,24]. Likewise, studies have shown that zymophagy, mediated by the VMP1-USP9x-p62 pathway, prevents PC cell death [54]. Consistently, the results of the present study revealed that muting autophagy marker LC3B resulted in significant decrease in the proliferation of cells that survive radiotherapy, implying the decisive role of autophagy in the activated cell survival of therapy-resistant PC cells.

Gemcitabine treatment complemented with cannabinoids induces ROS-mediated autophagic cell death in PC cells [55]. Radiation therapy and some forms of chemotherapy rely on ROS toxicity to eradicate tumor cells. Inhibition of autophagy or mitophagy increases ROS production, resulting in apoptotic cell death. To our knowledge, the present study is the first of its kind to recognize the potential of seaweed polyphenol fractions in the regulation of therapy-induced autophagy. Utilizing *in vitro* and clinically mimicking *in vivo* residual tumor models, the present study identified the autophagy-related molecular modulation that occurs after first-line radiotherapy, and further defined the clinical efficacy of high-polarity seaweed polyphenol fractions with demonstrated anti-PC potential. High basal autophagy in PC [18] is correlated with poor patient outcomes [19]. Intensified LC3 expression in PC tissue is associated with enhanced expression of carbonic anhydrase IX. More importantly, studies have shown that LC3 expression gradation is directly proportional to disease progression, in which absent → low → high → vesicular LC3 staining corresponds to low-grade PanIN-1 → PanIN-2 lesions → high-grade PanIN-3 → PDAC. Further, these studies suggested that autophagy contributes to PC progression. With this information, we investigated the expression levels of LC3A and LC3B in residual tumors after radiotherapy. Our findings clearly showed increased transcription of *LC3A* and *LC3B in vitro,* corresponding high levels of LC3A and LC3B in residual tumors *in vivo* and, demonstrate that muting LC3B significantly reduces cell proliferation in therapy resistant cells. New to science, this study demonstrate the LC3A and LC3B transcription and translation inhibitory potential of seaweed polyphenol fractions in surviving PC cells.

Conversely, studies have demonstrated that muting autophagy genes, including ATG5 and ATG3, significantly inhibits PC cell growth and colony formation of PDAC cell lines *in vitro* [16]. The results of the present study precisely delineated the benefits of SA-EA, PT-EA, and HT-EA seaweed polyphenol fractions in targeting radiotherapy-orchestrated LC3A and LC3B, and ATG3, ATG5, and ATG12 in this setting. Comprehensive inhibition of radiotherapy-orchestrated autophagy signaling by these polyphenols could identify unique drug-deliverables that could potentially impact PC treatment and yield better patient outcomes. HMGB1, a highly conserved protein containing HMG-box domains, regulates DNA replication, repair, recombination, and gene transcription. During stress, HMGB1 is released to the extracellular environment and functions as a DAMP [56]. The major receptors for HMGB1 are RAGE and TLR. Overexpression of HMGB1 and RAGE have been demonstrated in many tumors, including PC [57,58]. RAGE is a member of the IgG superfamily and plays important roles in the regulation of ROS generation and inflammatory response [59,60]. In PC, RAGE is compatible with PanIn formation [61]. Gemcitabine promotes RAGE expression by ROS-mediated NFκB activation [24]. This RAGE upregulation protects PC cells against oxidative injury and increases drug resistance by increasing autophagy and decreasing apoptosis [25].

The results of the present study showed increased transcriptional activation of HMGB1, RAGE, and the TLRs (TLRs1-9). As discussed above, though studies have demonstrated the activation of these molecules in PC, to our knowledge this is the first observation of elevated activation of these key molecules in PC cells that survive therapy. Studies have shown that silencing RAGE inhibits gemcitabine-, oxaliplatin-, or melphalan-induced autophagy by regulating Beclin 1-Vps34 interaction [25]. Consistently, our observations revealed a significant inhibition of HMGB1, RAGE, BECN1, and all TLRs in PC cells following treatment with seaweed polyphenol(s). As RAGE and TLRs are required for extracellular HMGB1-mediated autophagy [62], the cytosolic HMGB1 directly interacts with Beclin 1, which is controlled by the ULK1 complex [63,64]. Considering the comprehensive inhibition of these crucial players with the administration of seaweed polyphenols, we presume that this signal transduction flow through is involved in the polyphenol-associated inhibition of radiotherapy-induced autophagy.

Studies have also shown that autophagy functions as an anti-oncogenic mechanism in tumors [65-68]. It is believed that autophagy partly promotes cancer development through its ability to shape inflammatory reactions [9], metabolic requirements [10], and oxidative stress [11]. Thus, autophagy may play opposite roles in early and late stages of cancer development. One possible explanation for this difference is that the components of metabolic stress, the immune response, and the microenvironment are different. The inter-relationship between autophagy and apoptosis might decide tumor cells’ fate. In many cases, blockage of autophagy sensitizes tumor cells to anticancer therapy, including chemotherapy, radiation, and immunotherapy [69]. However, under certain conditions like apoptosis deficiency, autophagy can also be pro-death; this concept is termed “autophagic cell death” [70,71]. The notion of autophagic cell death was first established based on observations of increased autophagic markers in dying cells. An increasing number of studies indicate that autophagic cell death is cell death with autophagy, rather than cell death by means of autophagy [71,72].

The efficacy of autophagy inhibitors, including chloroquine or its derivatives, used in combination with chemotherapeutic drugs, radiation, and immunotherapy has been shown to inhibit acidification of the lysosome in preclinical models. This combination therefore prevents autophagy by blocking autophagosome fusion and degradation. Current ongoing clinical trials examining the benefit of autophagy inhibitors combined with gemcitabine in the treatment of PC are underway. These preliminary studies suggest that autophagy can contribute to PC progression. There is an immediate unmet need for deliverables that could target this signaling and response. The results of the present study identified three potential seaweed fractions that could serve as drug-deliverables against PC, particularly in the regulation of therapy-associated autophagy, and could prompt a significant archetypal shift in the treatment of PC patients.

## Conclusions

Taken together, in vitro (genetically diverse human Panc-3.27 and MiaPaCa-2 cells) and *in vivo* (clinically translatable residual PC mouse model) approaches coupled with clinically relevant fractionated radiation dose regimens, recognize the activation of autophagy signaling in radiotherapy resistant pancreatic cancer cells and the influence of autophagy on induced proliferation in surviving cells. In addition, well characterized (proven anti-tumorigenic) ethyl acetate polyphenol fractions from marine brown-algae, *Spatoglossum asperum* (SA-EA), *Padina tetrastromatica* (PT-EA), or *Harmophysa triquerta* (HT-EA) exerted anti-proliferation in therapy resistant PC cells. Further, the results presented in this study demonstrate that seaweed polyphenols prevent, as well as, target radiotherapy-activated autophagy signaling and associated radio-resistant cell proliferation. These data define the autophagy blueprint in therapy-resistant PC cells and further identify these polyphenols as adjuvants for potentiating current PC treatment modalities, radiotherapy here in this case, by targeting therapy orchestrated autophagy in residual cells. Further careful characterization of the compounds, their pharmacokinetic profiling, cytotoxicity profiling, *in vivo* bioavailability, and drug clearance evidence coupled with tumor-targeted delivery approaches would improve the outcomes of PC patients and, ultimately, could save innumerable lives.

## Abbreviations

PC: Pancreatic cancer
ATG: Autophagy related genes
SA: *Spatoglossum asperum*
PT: *Padina tetrastromatica*
HT: *Hormophysa triquerta*
EA: Ethyl acetate
Gy: Gray
TMA: Tissue microarray
IHC: Immunohistochemistry
H&E: Hematoxylin and eosin
WGA: Wheat germ agglutinin
FIR: Fractionated irradiation
PBS: Phosphate buffered saline
Ab: Antibody
BrdU: Bromodeoxyuridine
TLRs: Toll-like receptors
